# Genome Sequence of Classical Swine Fever Virus NIVEDI-165, Subtype 1.1, a Field Virus Strain Isolated from the Southern Part of India

**DOI:** 10.1128/MRA.00295-19

**Published:** 2019-05-23

**Authors:** S. S. Patil, K. P. Suresh, R. G. Amachawadi, David A. Meekins, J. A. Richt, Mainak Mondal, J. Hiremath, D. Hemadri, H. Rahman, Parimal Roy

**Affiliations:** a Virology Section, ICAR-National Institute of Veterinary Epidemiology and Disease Informatics (NIVEDI), Yelahanka, Bengaluru, India; b Disease Informatics Laboratory, ICAR-National Institute of Veterinary Epidemiology and Disease Informatics (NIVEDI), Yelahanka, Bengaluru, India; c Department of Clinical Sciences, College of Veterinary Medicine, Kansas State University, Manhattan, Kansas, USA; d Department of Diagnostic Medicine/Pathobiology (CEEZAD), College of Veterinary Medicine, Kansas State University, Manhattan, Kansas, USA; e Regional Representative for South Asia, ILRI, New Delhi, India; Portland State University

## Abstract

The whole-genome sequence of an Indian field isolate of classical swine fever virus, NIVEDI-165, was found to be subtype 1.1, and it showed 89 to 99% amino acid identity and 84 to 99% nucleotide identity with four and five Indian classical swine fever virus (CSFV) isolates, respectively. To the best of our knowledge, this is the first report on a full-genome sequence of CSFV from South India.

## ANNOUNCEMENT

Classical swine fever (CSF) is an economically important disease of pigs which causes 100% mortality in piglets, leading to disturbances in the economy of socially and economically disadvantaged people in India. Classical swine fever virus (CSFV), the causative agent, is a member of the genus Pestivirus within the family *Flaviviridae,* which also comprises bovine viral diarrhea and border disease of sheep viruses (1). CSFV is an enveloped virus containing a single-stranded nonsegmented positive-sense ∼12.3-kb-long RNA genome, which includes untranslated regions (UTRs) at both the 3′ and 5′ ends. CSFV has one serotype divided into three major genotypes (1 to 3) and 10 subgenotypes (1.1, 1.2, 1.3, 2.1, 2.2, 2.3, 3.1, 3.2, 3.3, and 3.4) (2). Phylogenetic analysis of Indian CSFV sequences indicated the continued dominance of subtype 1.1 (3, 4); however, detailed sequence analysis has shown the gradual emergence of subtype 2.2 (5–7).

Total RNA was extracted from the homogenate of spleen and liver samples collected from a pig infected with CSFV using an RNeasy Plus minikit (Qiagen, Inc., Germany). cDNA was synthesized using a RevertAid first-strand cDNA synthesis kit (Thermo Scientific) with random hexamers. cDNA was PCR amplified using primers specific for nine different overlapping regions of the CSFV genome, including the 5′ and 3′ termini, and cloned into a pJET1.2/blunt vector (Thermo Scientific). The recombinant plasmids were then sent to a commercial service (Bioserve India Pvt. Ltd., Hyderabad, India) for Sanger sequencing on an ABI Prism 3730 xl DNA sequencer. Twenty-eight sequence fragments were trimmed to remove vector-derived sequences, regions containing low Phred scores, and ambiguous base calls and were assembled with minimum 30-bp overlaps using CLC Genomics Workbench version 7 (CLC bio, Qiagen) to determine the full-genome sequence of 12,297 bp. The assembled genome had a GC content of 46% and encodes a polyprotein from nucleotide positions 374 to 12070 (5' UTR [nucleotides 1 to 373] and 3' UTR [nucleotides 12070 to 12297]) with an open reading frame (ORF) encoding a 3,898-amino acid polyprotein.

The resulting assembled genome was subjected to ClustalW sequence alignments and phylogenetic analysis against available CSFV genome sequences in the NCBI database using MacVector version 15. NIVEDI-165 clustered with other genotype 1.1 viruses in a phylogenetic analysis with 152 complete CSFV genome sequences. A total of nine whole-genome sequences of CSFV of Indian origin could be retrieved from the NCBI. The amino acid sequence of the NIVEDI-165 polyprotein had identity/similarity values with the Indian CSFV isolate sequences ranging from 89.0 to 99.7% for identity and 94.1 to 99.8% for similarity (GenBank accession numbers and identity/similarity values [%] are KM262189, 99.7/99.8; KC503764, 99.2/99.6; KY860615, 97.0/98.5; EU857642, 95.6/97.6; JQ861548, 92.0/96.4; KC533775, 91.2/95.7; KC533776, 91.4/96.0; KC533793, 90.8/95.5; and KC851953, 89.0/94.1). The nucleotide sequence of NIVEDI-165 had identity with the Indian CSFV isolate sequences ranging from 83.5 to 99.7% (GenBank accession numbers and identity values [%] are KM262189, 99.7; KC503764, 99.5; KY860615, 96.3; EU857642, 94.1; JQ861548, 84.9; KC533775, 84.5; KC533776, 84.5; KC533793, 84.3; and KC851953, 83.5).

In comparison to the sequence with GenBank accession number KC503764, the NIVEDI-165 sequence had 7 Ts at nucleotide position 12228 in the 3′ UTR region, and further analysis is needed to understand the importance of those Ts. The complete genome of NIVEDI-165 CSFV from Karnataka State, South India, and its comparison with other complete genomes of this region will help in understanding the epidemiology of circulating CSF viruses in this region in the future.

### Data availability.

The complete genome sequence of CSFV isolate NIVEDI-165 has been deposited in GenBank under the accession number MH734359.
